# Optimal artificial urine formulations for *in vitro* urolithiasis models

**DOI:** 10.3389/fruro.2026.1806726

**Published:** 2026-05-14

**Authors:** Deok Hyun Han, Jae Hoon Chung

**Affiliations:** 1 Department of Urology, Samsung Medical Center, Sungkyunkwan University School of Medicine, Seoul, Republic of Korea; 2 Department of Urology, Hallym University Sacred Heart Hospital, Hallym University College of Medicine, Anyang, Republic of Korea

**Keywords:** biofilms, *in vitro* techniques, salts, stents, urine

## Abstract

**Purpose:**

To develop and characterize standardized *in vitro* artificial urine (AU) models for three major stone-forming environments—calcium oxalate (CaOx), uric acid (UA), and infection-related struvite—and to identify assay operating conditions that maximize stent-associated surface deposition while limiting excessive bulk precipitation.

**Materials and methods:**

Homemade AU models were formulated from a physiologic base composition and modified to create disease-specific lithogenic conditions. The CaOx model used sodium oxalate fixed at 80 mg/L with stepwise calcium chloride escalation to 600%. The UA model was tested at uric acid concentrations of 500–1000 mg/L under acidic pH. The bacterial infection model was tested as two independent live-bacterial arms preset at bulk pH 6.5 or 9.0 before immersion to capture urease-driven alkalinization and surface colonization, and a non-bacterial ammonia-alkalinized control was also evaluated. Stents of three commercial types were immersed for 48 h under CDC biofilm-reactor conditions, and surface deposition was quantified as weight change per unit length (mg/mm).

**Results:**

In the CaOx model, surface-associated weight gain increased with calcium concentration up to 200% (0.98 g/L) but declined at higher levels owing to competing bulk precipitation. The UA model produced minimal deposition, with maximal weight gain at 600 mg/L. In the bacterial infection assay, weight gain was numerically highest in the arm preset at bulk pH 6.5 before immersion and remained substantial in the arm preset at pH 9.0, whereas the non-bacterial alkalinization model resulted in negligible deposits.

**Conclusions:**

These stone type–specific AU models provide defined, lab-preparable lithogenic screening conditions. Within this 48-h reactor assay, CaCl_2_ 200% + oxalate 80 mg/L, uric acid 600 mg/L, and the bacterial infection model initiated at preset bulk pH 6.5 yielded the highest observed stent-associated deposition. The platform may serve as an accelerated screening tool for evaluating stent materials, coatings, and anti-encrustation strategies.

## Introduction

Ureteral stents are used to maintain urinary tract drainage, either temporarily or for long-term palliative care, especially in patients with malignant ureteral obstruction (1). However, one of the most significant limitations of indwelling ureteral stents is encrustation, which results from mineral precipitation on the stent surface and is often associated with bacterial biofilm formation (2).

The degree of encrustation generally increases with indwelling time because prolonged residence exposes the stent surface to repeated cycles of crystal nucleation, bacterial colonization and biofilm maturation, and progressive mineral accretion (3). Stone formers are particularly prone to encrustation because supersaturated urine promotes crystal formation. Stents with higher encrustation are associated with infection, higher urine calcium, and prolonged indwelling duration (4). These pathological conditions represent a spectrum of metabolic or infectious risk profiles, leading to different types of urolithiasis, such as calcium oxalate (CaOx), uric acid (UA), or struvite stones (5).

Numerous innovations have been proposed for preventing or mitigating stent encrustation. However, *in vivo* evaluation is often impractical because of the high inter-individual variability in urine composition, ethical concerns, and cost. Therefore, reliable *in vitro* experimental systems are crucial for screening stent materials or coatings for encrustation resistance (6).

Despite this need, there are no widely adopted standardized artificial urine (AU) formulations that accurately simulate the diverse biochemical environments encountered in patients with stones. Most current AU models are based on healthy urine with near-neutral pH and balanced ionic profiles, which may not sufficiently challenge stents under high-risk stone-forming conditions (7–9).

In this study, we developed and optimized AU formulations to simulate three distinct stone-forming environments (CaOx, UA, and infection-related struvite) and to establish a short-term (48-h), accelerated *in vitro* platform for rapid screening of stent-surface deposition under lithogenic urine conditions. Using controlled CDC biofilm reactor conditions, we quantified surface deposition by gravimetric weight change per unit length and verified the prepared AU formulations by pre-immersion 24-hour urine analysis and urinalysis.

## Materials and methods

### Study design and protocol

**Figure 1:**
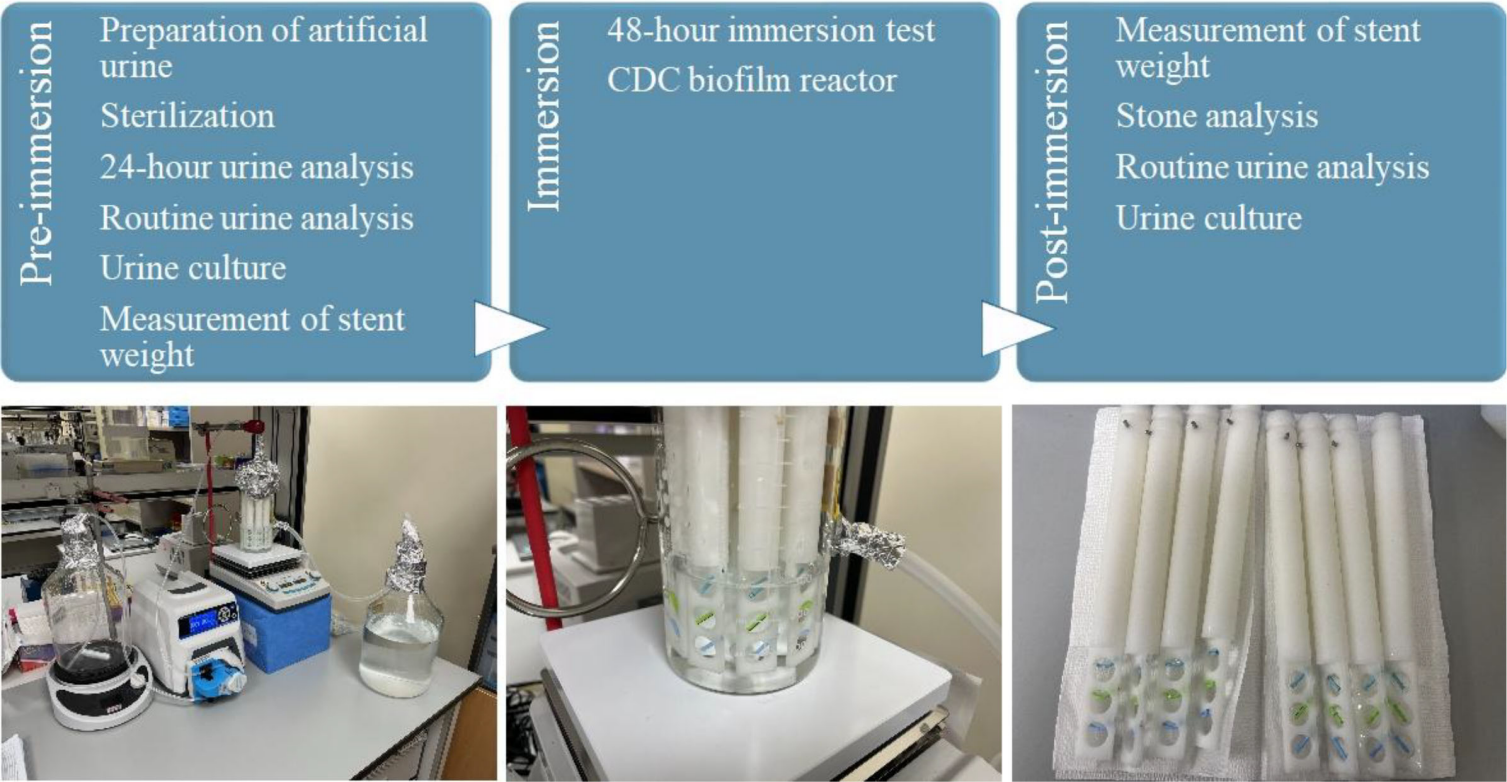
Flow sheet of the study and CDC biofilm reactor setting.

This *in vitro* study aimed to develop and optimize AU formulations to simulate three distinct stone-forming environments: CaOx, UA, and struvite stones. After confirming the intended pre-immersion chemistry of each formulation and sterility of the non-bacterial control solutions, ureteral stent segments were immersed in the respective AU models for 48 h in a CDC biofilm reactor (BioSurface Technologies Corp., Bozeman, MT, USA) (**Figure 1**). The primary endpoint was the identification of assay operating conditions that yielded the greatest stent-associated weight gain while avoiding excessive bulk precipitation. Additional analyses included stone composition profiling, pre-immersion urine verification, and adherent-bacteria assessment in the infection models.

### Artificial urine formulation and validation

The AU formulations were designed to replicate the key chemical and microbiological features of normal urine, CaOx, UA, and struvite stone-forming environments (6, 8, 10–12). For the lithogenic models, the goal was to establish disease-relevant supersaturation and alkalinization conditions for comparative stent screening rather than to reproduce average healthy urinary chemistry. All AU formulations were verified using 24-hour urine analysis prior to the immersion test.

#### Normal AU model

The normal AU composition included calcium chloride (0.49 g/L), magnesium chloride (5.5 g/L), sodium chloride (4.6 g/L), sodium sulfate (2.3 g/L), potassium dihydrogen phosphate (2.8 g/L), potassium chloride (1.6 g/L), ammonium chloride (1.0 g/L), urea (25 g/L), creatinine (1.1 g/L), sodium citrate (0.5 g/L), sodium oxalate (45 mg/L), and UA (250 mg/L). Each component was weighed and dissolved in distilled water with constant stirring. The pH of the normal AU was adjusted to 6.5 using sodium hydroxide.

#### CaOx AU models

The base CaOx AU composition was prepared by adding 35 mg/L sodium oxalate to the normal AU. Calcium chloride was then increased stepwise to 125% (0.6125 g/L), 150% (0.735 g/L), 200% (0.98 g/L), 300% (1.47 g/L), 400% (1.96 g/L), and 600% (2.94 g/L). These CaOx formulations were intentionally shifted into a supersaturated lithogenic range relative to the normal AU model to challenge stent surfaces under high-risk stone-forming conditions. The pH was adjusted to 6.5 using sodium hydroxide.

#### UA AU models

UA AU models used the normal AU formulation, which contained uric acid at 250 mg/L, as the baseline comparator. UA-specific AU models were then prepared by increasing uric acid to 500, 600, 750, and 1000 mg/L. The pH of the UA AU model was adjusted to 6.0 using sodium hydroxide.

#### Infection models

Proteus mirabilis (ATCC 29906; KCTC, Korea) was used to establish an infection-induced struvite stone model. Live P. mirabilis was selected to capture not only urease-mediated alkalinization but also bacterial adhesion and biofilm-related surface effects that are absent in a purified urease-only system. The strain was stored at −80 °C in glycerol stock until use. For activation, the stock was thawed and inoculated into 50 mL of Tryptone Soya Broth (TSB; Medion, Darwin Biotech, Korea), followed by incubation at 37 °C in a shaking incubator for 24 h. The bacterial culture was centrifuged at 4,000 rpm for 10 min to remove the supernatant, and the pellet was resuspended in TSB. The bacterial density was adjusted to 5×10^5 CFU/mL using a Densicheck device, and 10 mL of the final inoculum was used for the experiments. Bacterial inoculum (10 mL) was introduced into the secondary chamber of the reactor. Two independent live-bacterial infection arms were prepared by presetting the bulk pH of the AU to 6.5 or 9.0 before immersion. The same base AU formulation, P. mirabilis strain, and inoculum density were used in both arms. No pH-stat system was used, and endpoint pH after the 48-hour immersion was not formally monitored. Only the bacterial infection arms received live inoculum; the normal, CaOx, UA, and ammonia-alkalinized comparator arms were maintained as non-bacterial controls.

To develop a non-bacterial comparator, an ammonia solution (1.2 M) was added to the standard AU to mimic alkalinization in the absence of live bacteria. This arm was intended as a mechanistic alkalinization control rather than a fully physiologic infection surrogate. The prepared comparator solution underwent pre-immersion urine verification, and during reactor operation the bulk pH was adjusted to approximately 6.33 to maintain flow stability and avoid premature bulk precipitation.

### Ureteral stent preparation

Three types of ureteral stents were tested in this study: the Tria™ Soft Ureteral Stent (Boston Scientific, Marlborough, MA, USA), Inlay Optima Ureteral Stent™ (BD, Covington, GA, USA), and Endo-Sof™ Double Pigtail Stent (Cook Medical, Bloomington, IN, USA). All stents were 6 Fr, 30 cm in length, and cut into eight 15-mm segments (two from each coil end and four from the shaft) to fit the CDC reactor holders, standardize geometric exposure, and enable per-length comparison under identical reactor conditions. In preliminary bench handling before the formal experiment, segment cutting did not produce gross visible coating disruption that would preclude comparative testing. After rinsing with sterile distilled water to remove manufacturing residue and handling debris before baseline weighing, the segments were desiccated at room temperature for 24 h and then weighed using an analytical balance. The segments were mounted on custom holders and sterilized using ethylene oxide (E.O.) gas prior to use.

### Immersion test using CDC biofilm reactor

Immersion tests were performed in a CDC biofilm reactor. For each condition, the designated AU formulation was prepared fresh as a single premixed solution and delivered through one sterile line to the reactor at a flow rate of 1 mL/min. Continuous agitation was maintained within the reactor chamber during immersion; separate calcium- and oxalate-containing reservoirs, in-line mixing immediately before chamber entry, or additional upstream anti-precipitation measures were not used. This simplified configuration was intentionally used to identify a practical operating range, including the concentration boundary at which bulk precipitation begins to compete with surface deposition. Each stent fragment was suspended within the reactor, and agitation was maintained at 60 rpm and 37 °C throughout the 48-hour immersion period. The tubing and filters were sterilized with E.O., and the reactor components were autoclaved. The final assembly and experimental procedures were conducted in a UV-irradiated laminar flow cabinet to ensure aseptic conditions (**Figure 1**).

### Post-immersion evaluation

Following immersion, the stents were carefully removed and rinsed with sterile distilled water. They were then dried in a desiccator for 24 h and weighed again under identical conditions. The degree of surface deposition was calculated as the difference between the pre- and post-immersion weights using a precision analytical balance. Because gravimetric weight gain can reflect both retained mineral deposits and adherent biomass, this endpoint was interpreted as total surface-associated deposition rather than pure mineral mass. Stone-like deposits adherent to the stent surface were analyzed using Fourier-transform infrared spectroscopy to determine their crystalline compositions. **Supplementary Table S1** summarizes the pre-immersion 24-hour urine analysis and urinalysis used to verify the prepared AU formulations. Formal endpoint pH after the 48-hour immersion was not monitored, and local pH at the stent-biofilm interface was not directly measured. In the infection models, after the 48-hour immersion period, the stents were removed and rinsed three times with sterile PBS to eliminate non-adherent planktonic bacteria before the adherent-bacteria recovery assay. Each stent was then placed in 1 mL of PBS and ultrasonicated for 15 min, followed by vortexing for 30 s. The resulting bacterial suspension was serially diluted (1:100) and spread onto TSA plates. After incubation at 37 °C for 24 h, the bacterial colonies were counted to quantify bacterial adherence to the stent surfaces (**Figure 1**).

## Results

Pre-immersion 24-hour urine analyses and urinalysis confirmed that the chemical compositions of the prepared AU samples corresponded to the intended formulations (**Supplementary Table S1**).

### Normal AU model

Urinalysis and culture confirmed sterility; encrustation was negligible (<0.01 mg/mm) (**Table 1**). Because the amount of deposit formed was below the analytical threshold, stone analysis of the crystalline composition was not performed.

**Table 1:** Weight change per unit length.

Stent	Pre-immersion			Post-immersion			
	Length	Weight	Weight/length	Weight	Weight/length	Weight difference	Weight difference/length
Boston (n=8)	15.10[14.99 - 15.19]	39.55[39.10 - 39.98]	2.62[2.60 - 2.64]	39.75[39.33 - 40.08]	2.64 [2.61 - 2.64]	0.15[0.03 - 0.30]	0.01[0.00 - 0.02]
BD (n=8)	14.85[14.75 - 15.06]	38.90[37.83 - 39.60]	2.62[2.53 - 2.68]	39.15[38.30 - 40.25]	2.64[2.54 - 2.71]	0.15[0.00 - 0.65]	0.01[0.00 - 0.04]
Cook (n=8)	14.80[14.77 - 14.83]	43.15[42.60 - 44.58]	2.91[2.87 - 3.02]	43.25[42.63 - 44.75]	2.92[2.88 - 3.03]	0.05[0.00 - 0.10]	0.00[0.00 - 0.01]

### CaOx AU models

**Figure 2:**
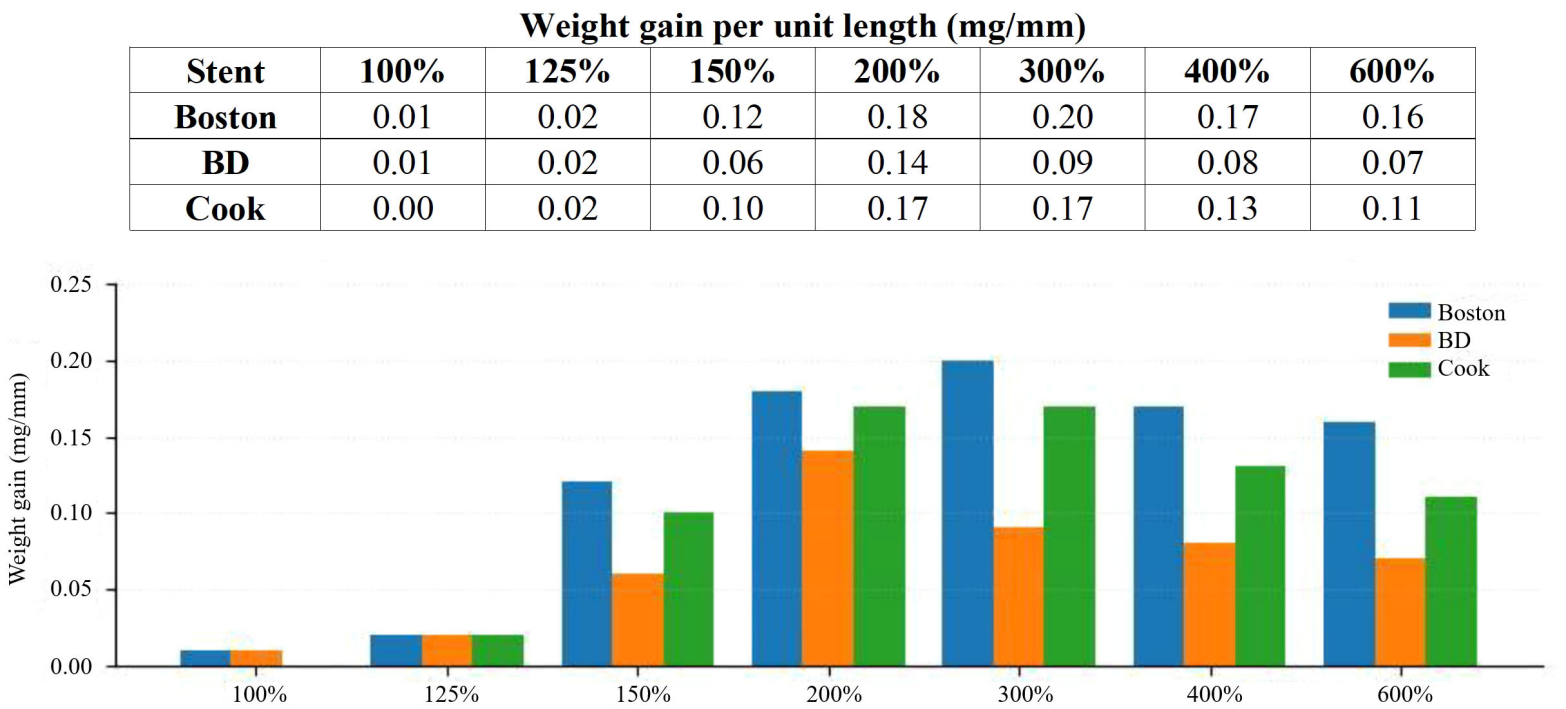
CaOx AU models.

Quantitative evaluation of surface deposition revealed a clear concentration-dependent pattern of weight gain per unit stent length across the different CaCl_2_ concentrations. Surface-associated weight gain peaked at 200% CaCl_2_ (~0.20 mg/mm) across all stent types, then declined at higher concentrations (≥300%), consistent with a shift toward competing bulk precipitation (**Figure 2**). FTIR analysis of the deposits formed in the CaOx AU models confirmed the presence of CaOx crystals.

### UA AU models

**Figure 3:**
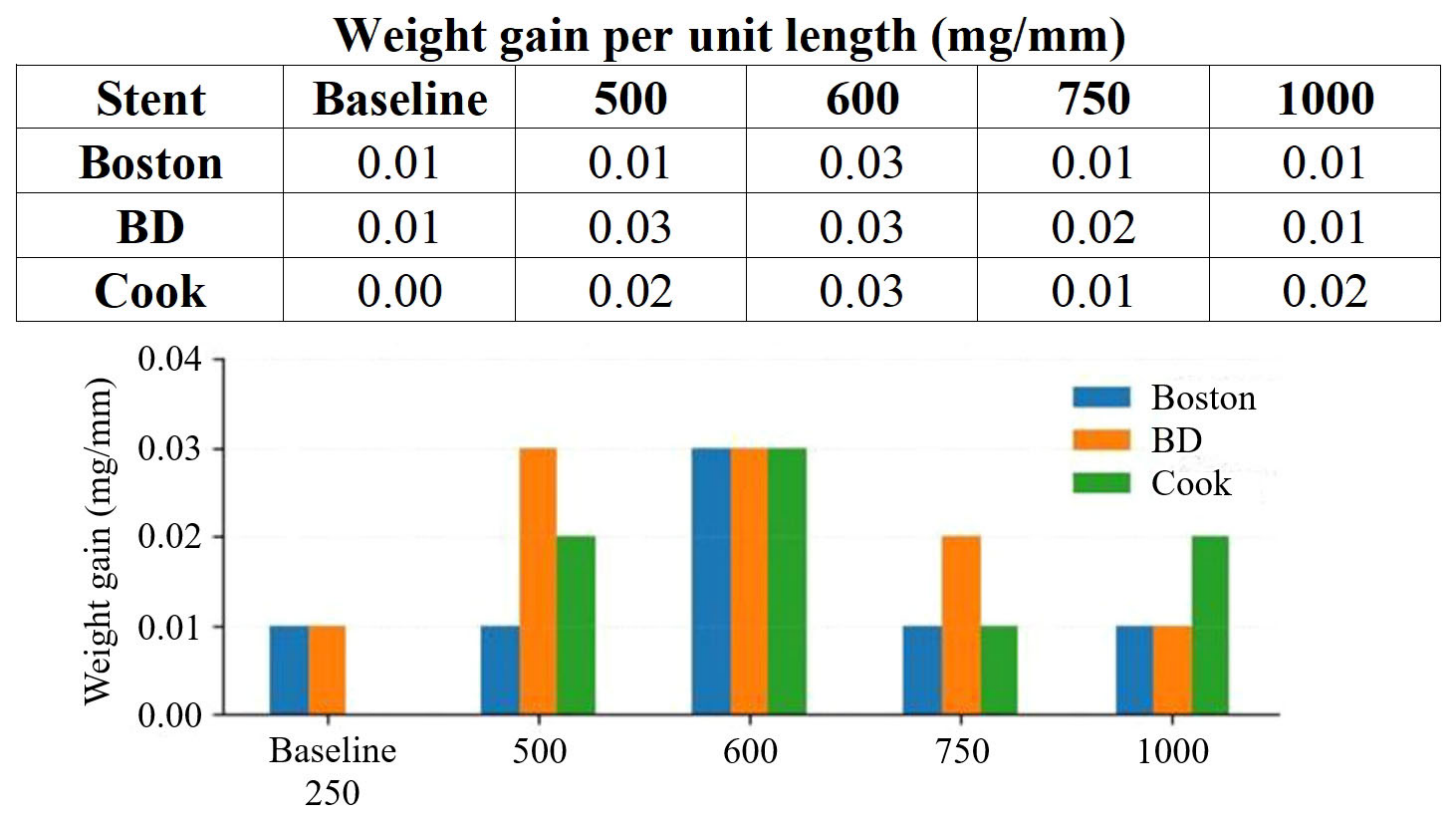
Uric acid AU models.

All stent types showed minimal surface deposition (<0.03 mg/mm), with a small peak at 600 mg/L UA (**Figure 3**). The 250 mg/L value shown in **Figure 3** represents the normal AU baseline comparator rather than an additional UA-specific model. FTIR analysis of the deposits in the UA AU models confirmed UA crystals.

### Infection models

In the non-bacterial alkalinization control, the prepared comparator solution was verified before immersion (**Supplementary Table S1**). During reactor operation, ammonia-induced pH elevation led to visible precipitation when the bulk pH approached 6.8. Therefore, the bioreactor was operated at an adjusted bulk pH of approximately 6.33 in this arm to maintain flow stability and avoid premature bulk precipitation. Under these conditions, weight gain per unit length was 0.01 mg/mm or less across all stent types.

**Figure 4:**
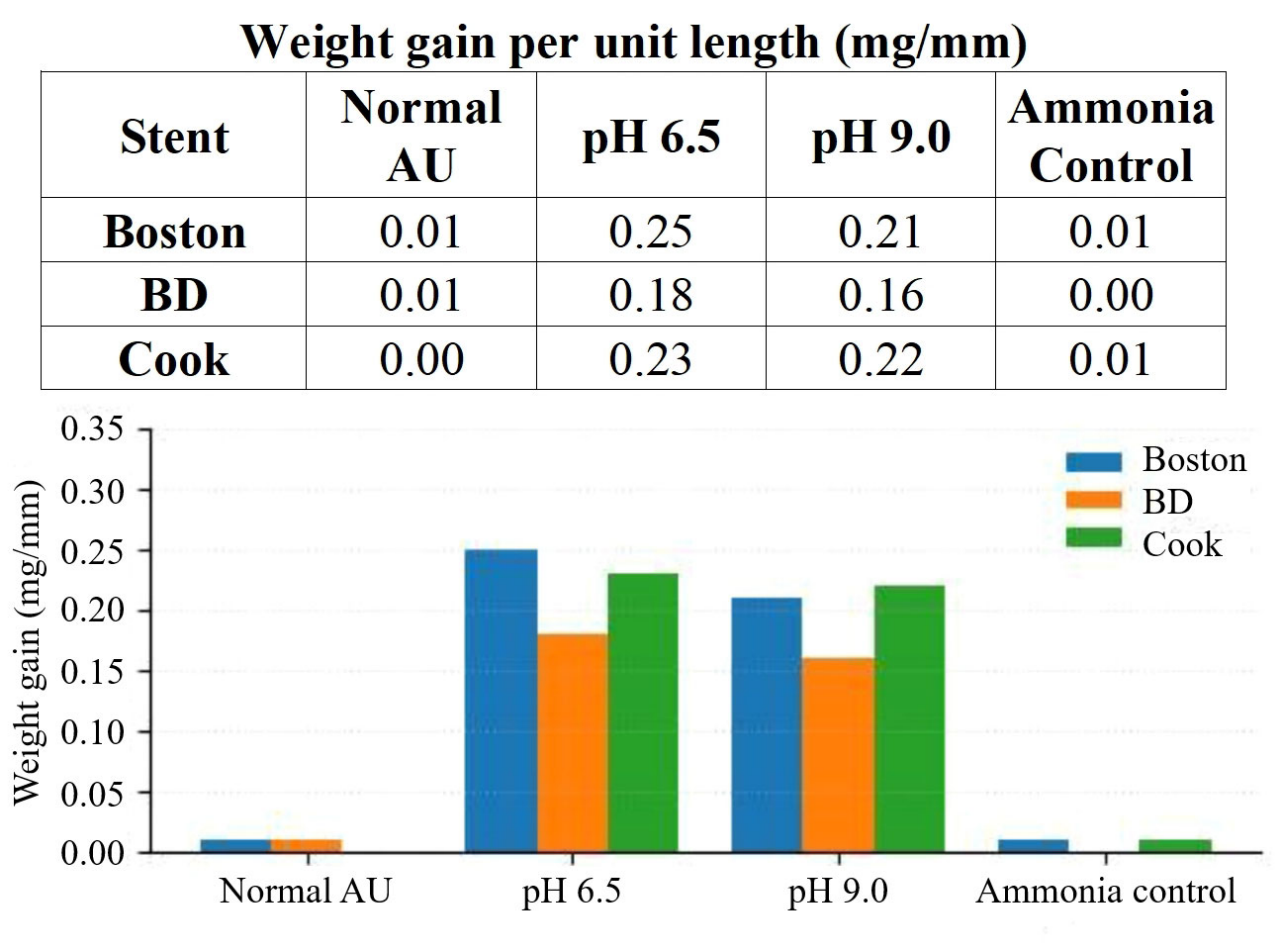
Infection AU models.

In the bacterial infection AU models prepared at preset bulk pH 6.5 and preset bulk pH 9.0 before immersion, surface-associated weight gain was numerically highest in the preset pH 6.5 arm (~0.25 mg/mm) and remained substantial in the preset pH 9.0 arm (**Figure 4**). More visible bulk precipitation was also observed in the bottle/reservoir of the preset pH 9.0 arm. FTIR analysis of deposits recovered from the bacterial infection arm demonstrated struvite-predominant crystals. Because formal inferential comparison and systematic side-by-side spectral comparison between the pH 6.5 and pH 9.0 conditions were not performed, these findings should be interpreted as assay-specific observations rather than evidence that pH 6.5 is the universal biochemical optimum for struvite formation.

## Discussion

In this study, we developed standardized, lab-preparable AU models for three distinct stone-forming environments and evaluated them as an accelerated *in vitro* screening platform for stent-surface deposition.

Standardized AU systems are needed because current formulations, mostly based on healthy urine, may not sufficiently challenge stents under high-risk stone-forming conditions (13, 14). Commercial AU products also exhibit inconsistent composition. Although existing AU formulations have been useful, most are designed to mimic healthy urine with near-neutral pH and balanced ionic profiles. Our models aimed to fill this gap by providing AU compositions tailored to disease-predisposing urinary milieus. To date, the literature has not presented well-defined, broadly applicable operating conditions for short-term comparative stent screening. Therefore, in this study, we used defined, lab-preparable methods and deliberately characterized the transition from surface retention to bulk precipitation. These challenge conditions are deliberately accelerated and should not be interpreted as direct one-to-one physiologic equivalents of average clinical urine.

As expected, the normal AU model did not produce meaningful encrustation, and urinalysis and culture confirmed the expected chemical and microbiological behavior of the system. This highlights the limitation of healthy-urine AU for discriminating stent performance under high-risk stone-forming or infected conditions. At the same time, our lithogenic models should be understood as deliberately accelerated and more challenging than average clinical urine, rather than as direct one-to-one physiologic equivalents. Moreover, during model development, we observed that when solute concentrations exceeded certain thresholds, oversaturation of the AU led to bulk salt precipitation rather than encrustation on the stent surfaces, confirming that such conditions are unsuitable for stent studies.

The 48-h immersion duration was selected *a priori* to establish a rapid, standardized, and discriminative screening platform for lithogenic AU optimization and material-surface evaluation. It was not intended to reproduce the clinical calendar time course of stent dwell time in humans. A short experimental window helps minimize time-dependent confounders that can obscure surface-encrustation readouts, including progressive ionic depletion, pH drift, extensive bulk precipitation in the surrounding solution, and, in infection models, uncontrolled bacterial overgrowth.

### CaOx models: concentration-dependent crystallization

Kavanagh et al. quantified the changes in the crystallization rate at various concentrations while maintaining a constant Ca–Ox ratio in AU, demonstrating that supersaturation is the dominant factor governing the precipitation behavior (10). However, AU has been reported to suppress the precipitation rate of CaOx owing to its ionic strength and specific inhibitory components, suggesting that subtle differences in the medium composition, rather than simply higher concentrations, influence the crystallization process (15). In the present study, we similarly confirmed that high levels of supersaturation promoted precipitation; however, such conditions were unfavorable for stent encrustation. Excessively rapid precipitation resulted in extensive crystallization within the solution itself rather than on the stent surface, indicating that these conditions are unsuitable for stent-based AU experiments. Previous studies did not clearly present quantitative data or operational thresholds describing the inverse relationship, in which increased bulk precipitation at high concentrations led to decreased actual deposition on the stent surface.

More recently, a study comparing stent materials in a lithogenic AU environment reported that the AU composition significantly affects stent encrustation profiles (13). To overcome this limitation, we used three representative commercially available stents of each AU composition. Although the degree of encrustation varied among stent types, the AU composition that induced the greatest stent deposition was consistent across all stents.

In earlier studies, systematic pre-immersion verification of AU chemical behavior and standardized microbiological control were rarely described. To address this gap, our study established a consistent framework for refining the CaOx AU model—sequential oxalate concentration screening, stepwise CaCl_2_ increment, and weight change per unit stent length—to define a practical experimental operating point.

During preliminary optimization, we found that increasing sodium oxalate up to 80 mg/L elevated oxalate concentrations without affecting Ca²^+^ levels or turbidity, whereas concentrations above 80 mg/L led to increased turbidity and a paradoxical decrease in the measured Ca²^+^, indicating the onset of bulk precipitation. Based on this, oxalate was fixed at 80 mg/L, and stepwise increases in CaCl_2_ revealed that weight gain per unit length peaked at 200% CaCl_2_ (0.98 g/L), with higher concentrations (≥300%) reproducing an inverse relationship in which surface deposition declined as competing bulk precipitation increased. Thus, the CaOx model was intentionally driven into a supersaturated lithogenic range relative to the normal AU baseline; this was a design feature of the assay rather than an attempt to reproduce average physiologic urine chemistry. This phenomenon is consistent with the nonlinear kinetics of calcium oxalate crystallization described in prior studies (10, 16).

This study clearly defined a practical operating point applicable to stent research and delineated a zone in which bulk precipitation predominates over surface attachment. Such an approach is expected to enhance the sensitivity of coating and material screening and improve inter-study comparability in future AU-based encrustation research.

### UA models: limited encrustation due to solubility constraints

UA stones are a representative pathology associated with low urinary pH, hyperuricemia, and low urine volume. Several studies have explored this condition by using AU systems. Grases et al. reported that theobromine delayed the nucleation and growth of UA crystals (17). Another study demonstrated that pre-incubation of UA and monosodium urate in urine affects CaOx precipitation, suggesting that UA crystals may play a mediating role in the aggregation of CaOx particles (18). Nevertheless, studies focusing on stent encrustation under UA–specific conditions are extremely limited. Most previous studies have focused on identifying factors that promote or inhibit crystallization rather than quantifying stent encrustation or optimizing experimental operating points through variations in UA concentration or stent materials. Moreover, many of these studies employed formulations based on normal AU rather than reproducing the pathophysiological environment of UA stone formers, such as low pH, high UA levels, and low urinary output.

In the present study, we addressed these limitations by systematically adjusting UA concentrations and performing pre-immersion urinalysis and 24-hour urine chemistry analysis to ensure the intended biochemical characteristics of our AU compositions. However, the UA AU model yielded minimal encrustation. A modest increase was observed at 600 mg/L UA; however, higher concentrations led to bulk precipitation in the solution, limiting the deposition on the stent surface. The limited encrustation burden precluded detailed compositional analysis of most samples. These findings reflect the physicochemical characteristics of UA, which has poor solubility in acidic urine, leading to rapid crystallization in the bulk phase rather than on device surfaces.

### Infection models: strong encrustation driven by bacteria

Infection-related urinary stone formation is typically driven by urease-producing bacteria, such as Proteus mirabilis, which increase urinary pH and promote the precipitation of struvite and calcium phosphate crystals. The characterized structure of P. mirabilis biofilms grown in AU confirms that microbial urease activity is a key determinant of crystal deposition (19). A novel stent material with antibacterial and anti-encrustation properties demonstrates ongoing efforts to mitigate infection-associated encrustation (20). In this study, we used both P. mirabilis-inoculated and non-bacterial infection models. The latter served as a mechanistic alkalinization control and resulted in minimal surface deposition. We selected live P. mirabilis to integrate urease activity with bacterial attachment and biofilm-related surface effects that would not be captured by purified urease alone. At the same time, because a purified urease-only comparator was not included and biofilm architecture was not directly imaged, the bacterial arm should be interpreted as a pragmatic live-bacteria screening model rather than a reductionist urease-only system. The bacterial pH 6.5 and pH 9.0 arms were independently prepared preset bulk-pH conditions before immersion, not the beginning and endpoint pH values of a single experiment. **Supplementary Table S1** therefore summarizes pre-immersion urine verification rather than formal post-immersion endpoint chemistry. Within this setup, the markedly lower soluble calcium, magnesium, and phosphate observed in the preset pH 9.0 bacterial arm are most consistent with premature bulk precipitation under this highly alkaline starting condition. This interpretation is consistent with our experimental observation that the preset pH 9.0 arm showed greater visible bottle/reservoir precipitation and relatively less retained stent deposition than the preset pH 6.5 arm. The observation that surface deposition was numerically higher at preset bulk pH 6.5 than at preset bulk pH 9.0 should therefore be interpreted as an assay-specific operating point at which retained stent deposition exceeded competing bulk precipitation, not as a general statement that pH 6.5 is optimal for struvite chemistry. Moreover, no pH-stat system or formal endpoint pH monitoring was used, and the local pH at the stent-biofilm interface was not directly measured.

This study has several limitations. Some AU conditions yielded insufficient deposits for full compositional characterization. The 48-h platform is an accelerated screening assay and does not reproduce the full clinical dwell-time course of indwelling stents. Gravimetric weight gain was used as a surface-deposition endpoint, but mineral and bacterial biomass contributions were not analytically separated, biofilm architecture was not imaged, and a purified urease-only comparator was not tested. In addition, experiments were performed on cut stent segments rather than intact stents; although this enabled standardized reactor mounting and per-length comparison, it does not fully reproduce whole-stent geometry, may introduce edge-related artifacts, and cannot directly represent *in vivo* whole-stent behavior. Segment-based CDC testing has nevertheless been practically useful for comparative *in vitro* encrustation studies, including our prior reactor model (6, 21). Formal between-run reproducibility testing, comprehensive correlation of post-run bacterial burden with deposition, formal endpoint pH monitoring, real-time local surface pH monitoring, and inferential statistical comparison between the preset bacterial pH 6.5 and pH 9.0 conditions were not performed. The AU models also do not incorporate proteins, organic matrix components, or immune factors. However, the significance of this study lies in defining stone type-specific AU conditions that can support comparative *in vitro* research.

## Conclusions

The present study demonstrates the feasibility of tailored AU models that reflect distinct lithogenic environments in an accelerated *in vitro* stent-screening platform. Defined operating conditions were identified for the CaOx, UA, and preset-pH bacterial infection models. These formulations may serve as defined comparative benchmarks for the preclinical evaluation of stent materials, coatings, and anti-encrustation strategies as accelerated challenge conditions rather than direct one-to-one physiologic equivalents, while future work should incorporate independent repeat runs, biofilm imaging, and direct partitioning of biomass versus mineral deposition.

## Data Availability

The original contributions presented in the study are included in the article/**Supplementary Material**. Further inquiries can be directed to the corresponding author.

## Supplementary material

Supplementary Table 1 Pre-immersion 24-hour urine analysis and urinalysis of the prepared AU formulations.
