# Supplementary material for: Optimal artificial urine formulations for in vitro urolithiasis models
Source: Front Urol. 2026 May 14;6:1806726. doi: 10.3389/fruro.2026.1806726 (PMC13215919; doi:10.3389/fruro.2026.1806726)
Supplement: Supplementary Table 1 — Pre-immersion 24-hour urine analysis and urinalysis of the prepared AU formulations. [file Table1.docx]

| ​Supplementary 1. 24-hour urine analysis and urine analysis results​ | | | | | | | | | | | | | | | |
| --- | --- | --- | --- | --- | --- | --- | --- | --- | --- | --- | --- | --- | --- | --- | --- |
| Analysis | Normal AU | CaOx model | | | | | |  | Uric acid model | | | |  | Infectious model | |
|  |  | CaCl2 125% | CaCl2 150% | CaCl2 200% | CaCl2 300% | CaCl2 400% | CaCl2 600% |  | Uric acid 500mg | Uric acid 600mg | Uric acid 750mg | Uric acid 1000mg |  | Bacterial model | non-bacterial model |
| **24-hour urine analysis** |  |  |  |  |  |  |  |  |  |  |  |  |  |  |  |
| Calcium (Ca)(24hr U) | 154 | 199 | 214 | 250 | 313 | 621 | 771 |  | 179 | 164 | 176 | 171 |  | 50 | 166 |
| Chloride(Cl)(24hr U) | 183 | 182 | 182 | 209 | 183 | 206 | 232 |  | 177 | 175 | 179 | 175 |  | 246 | 187 |
| Magnesium (Mg)(24hr U) | 40.5 | 53.4 | 53.4 | 53.4 | 53.4 | 53.4 | 53.4 |  | 53.4 | 53.4 | 53.4 | 53.4 |  | 2.6 | 53.4 |
| Phosphate(P)(24hr U) | 0.65 | 0.62 | 0.62 | 0.45 | 0.62 | 0.62 | 0.50 |  | 0.65 | 0.65 | 0.66 | 0.65 |  | 0.03 | 0.03 |
| Potassium(K)(24hr U) | 43 | 42 | 43 | 44 | 44 | 42 | 45 |  | 42 | 44 | 44 | 43 |  | 55 | 43 |
| Sodium(Na)(24hr U) | 119 | 134 | 135 | 138 | 132 | 131 | 131 |  | 122 | 126 | 125 | 133 |  | 172 | 127 |
| BUN(24hr U) | 11.9 | 10.7 | 11.7 | 11.9 | 11.3 | 11.7 | 11.3 |  | 12.2 | 11.7 | 11.8 | 12.3 |  | 10.8 | 11.9 |
| Creatinine(24hr U) | 1.01 | 0.95 | 1.02 | 1.01 | 1.03 | 1.06 | 0.96 |  | 1.04 | 1.02 | 1.05 | 1.08 |  | 1.02 | 1.05 |
| Uric Acid(24hr U) | 175 | 138 | 121 | 158 | 149 | 121 | 103 |  | 102 | 583 | 712 | 975 |  | 114 | 255 |
| Oxalate | 25.5 | 28.6 | 40 | 34.1 | 35.2 | 59.4 | 23.2 |  | 31.2 | 31.2 | 31.3 | 32.2 |  | 85.8 | 34.8 |
| Citrate | 20 | 311 | 297 | 297 | 315 | 319 | 232 |  | 284 | 289 | 311 | 276 |  | 258 | 293 |
| **Urine analysis** |  |  |  |  |  |  |  |  |  |  |  |  |  |  |  |
| Specific gravity | 1.015 | 1.019 | 1.019 | 1.017 | 1.019 | 1.02 | 1.021 |  | 1.019 | 1.019 | 1.02 | 1.019 |  | 1.023 | 1.018 |
| Nitrite | Neg | Neg | Neg | Neg | Neg | Neg | Neg |  | Neg | Neg | Neg | Neg |  | Neg | Neg |
| pH | 6.5 | 6 | 6 | 6 | 5.5 | 6 | 5.5 |  | 5 | 6 | 6 | 6.5 |  | 9 | 6.5 |
| Protein | Neg | Neg | Neg | Neg | Neg | Neg | Neg |  | Neg | Neg | Neg | Neg |  | Trace | Neg |
| Glucose | Neg | Neg | Neg | Neg | Neg | Neg | Neg |  | Neg | Neg | Neg | Neg |  | Neg | Neg |
| Ketones | Neg | Neg | Neg | Neg | Neg | Neg | Neg |  | Neg | Neg | Neg | Neg |  | Neg | Neg |
| Urobilinogen | Norm | Norm | Norm | Norm | Norm | Norm | Norm |  | Norm | Norm | Norm | Norm |  | Norm | Norm |
| Bilirubin | Neg | Neg | Neg | Neg | Neg | Neg | Neg |  | Neg | Neg | Neg | Neg |  | Neg | Neg |
| Occult Blood | Neg | Neg | Neg | Neg | Neg | Neg | Neg |  | Neg | Neg | Neg | Neg |  | Neg | Neg |
| Urine WBC | Neg | Neg | Neg | Neg | Neg | Neg | Neg |  | Neg | Neg | Neg | Neg |  | Neg | Neg |
|  | | | | | | | | | | | | | | | |
